# Trends in High-Acuity Cardiovascular Events During the COVID-19 Pandemic

**DOI:** 10.1001/jamahealthforum.2023.4572

**Published:** 2024-01-05

**Authors:** J. Franklin Wharam, Robert F. LeCates, Ann Thomas, Fang Zhang, Stephanie Argetsinger, Laura F. Garabedian, Alison A. Galbraith

**Affiliations:** 1 Department of Medicine, Duke University, Durham, North Carolina; 2 Duke-Margolis Center for Health Policy, Durham, North Carolina; 3 Department of Population Medicine, Harvard Pilgrim Healthcare Institute, Harvard Medical School, Boston, Massachusetts; 4 Department of Pediatrics, Boston Medical Center and Boston University Chobanian and Avedisian School of Medicine, Boston, Massachusetts

## Abstract

This cohort study describes changes in myocardial infarction and stroke hospitalizations as well as congestive heart failure, angina, and transient ischemic attack incidents months before and after March 2020 among insured people in New England.

## Introduction

The COVID-19 pandemic and subsequent public health measures reduced health care access, potentially delaying time-sensitive cardiovascular disease (CVD) care. Additionally, SARS-CoV-2 infection is associated with higher rates of heart failure, myocardial infarction, and stroke.^1^ Population-level analyses reported early reductions in major adverse cardiovascular events (CVEs).^2,3,4,5^ This pattern raised concerns that subsequent rates might increase beyond expected levels, but longer-term patterns are uncertain. New England was among the earliest- and hardest-hit regions during the pandemic. Analyses of New England populations could provide insights about longer-term pandemic implications for CVD outcomes. We hypothesized that early decreases in adverse CVEs were followed by rates that were higher than those estimated by prepandemic CVE trends.

## Methods

We studied administrative and claims data from March 2017 to December 2021 from Harvard Pilgrim Health Care, a New England insurer with approximately 1 million members. We included commercial insurance and Medicare Advantage members 35 years or older from Massachusetts, New Hampshire, Maine, and Connecticut with at least 6 enrollment months (eTables 1-5 in Supplement 1). Harvard Pilgrim Health Care Institutional Review Board approved this cohort study and waived informed consent because deidentified data were used. We followed the STROBE reporting guideline.

Using claims-based algorithms and a standard approach with high specificity, we measured myocardial infarction and stroke hospitalizations (eMethods and eTable 6 in Supplement 1). We captured congestive heart failure (CHF), angina, and transient ischemic attack (TIA) episodes in individuals presenting to the emergency department, observation unit, or hospital. We added up the preceding measures to create a composite high-acuity CVE outcome. With an interrupted time series design, we examined monthly event rates before and after March 2020. We ran segmented linear regression models adjusted for age and sex to compare modeled postpandemic trends with expected postpandemic trends. Statistical analysis was performed with Stata 16 (StataCorp LLC).

## Results

Member characteristics were similar across the study period, but members had higher socioeconomic status (SES) than state populations. Composite high-acuity CVEs initially decreased in April 2020 by 26.6% (95% CI, −31.4 to −21.8) (Figure, Table). Rates remained below expected levels in March 2021 (−9.6%; 95% CI, −14.5 to −4.8) and December 2021 (−19.8%; 95% CI, −26.2 to −13.5).

**Figure.**
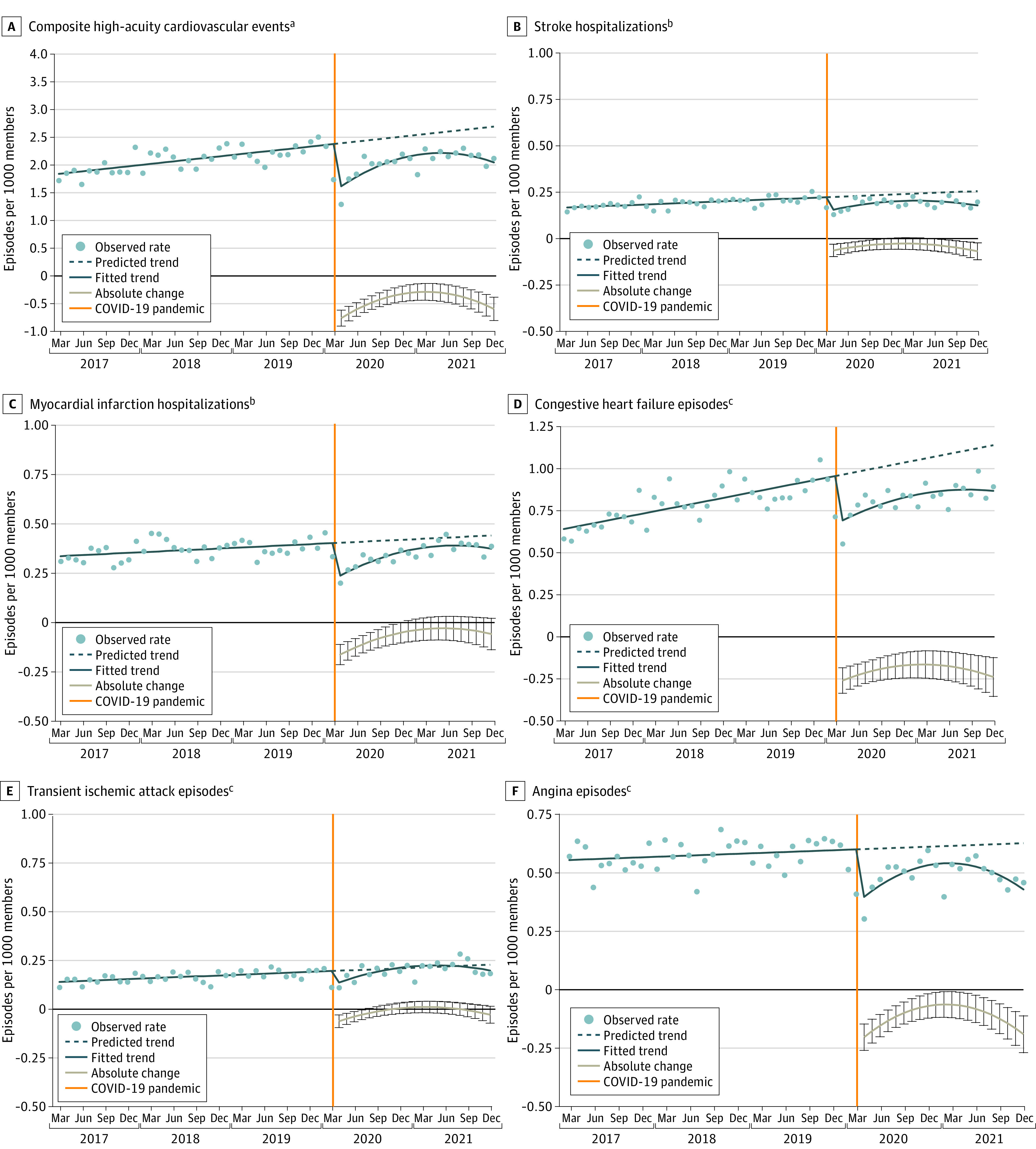
Rates per Month of Cardiovascular Events in a Commercially Insured Population 35 Years or Older The eMethods in Supplement 1 detail the segmented regression models used to generate absolute difference estimates with 95% CIs (represented by gray line with error bars) and observed monthly rates and trends. ^a^Comprising stroke, myocardial infarction, congestive heart failure, transient ischemic attack, and angina episodes. ^b^Presenting to the hospital. ^c^Presenting to the emergency department, observation unit, or hospital.

**Table.** Absolute and Relative Differences vs Expected Rates in High-Acuity Presentations for Adverse Cardiovascular Events During the COVID-19 Pandemic

Event	Difference vs expected rates (95% CI)				
	April 2020		Smallest relative difference [month of smallest difference during follow-up period]	December 2021	
	Absolute, episodes per 1000 members	Relative, %		Absolute, episodes per 1000 members	Relative, %
Composite high-acuity cardiovascular events^a^	−0.75 (−0.90 to −0.61)	−26.6 (−31.4 to −21.8)	−9.6 (−14.5 to −4.8) [March 2021]	−0.59 (−0.80 to −0.37)	−19.8 (−26.2 to −13.5)
Stroke^b^	−0.07 (−0.10 to −0.03)	−27.0 (−39.5 to −14.5)	−11.8 (−23.1 to −0.5) [February 2021]	−0.07 (−0.12 to −0.03)	−27.3 (−42.4 to −12.2)
Myocardial infarction^b^	−0.16 (−0.22 to −0.11)	−27.8 (−35.8 to −19.8)	−5.1 (−14.8 to 4.5) [June 2021]	−0.06 (−0.14 to 0.02)	−10.0 (−22.4 to 2.5)
Congestive heart failure^c^	−0.26 (−0.34 to −0.18)	−26.1 (−33.3 to −18.9)	−15.8 (−22.6 to −9.0) [March 2021]	−0.24 (−0.36 to −0.12)	−22.1 (−31.5 to −12.7)
Transient ischemic attack^c^	−0.06 (−0.09 to −0.03)	−26.7 (−40.6 to −12.9)	6.2 (−7.2 to 19.6) [March 2021]	−0.03 (−0.07 to 0.02)	−10.6 (−28.0 to 6.8)
Angina^c^	−0.20 (−0.26 to −0.14)	−26.2 (−33.0 to −19.4)	−8.0 (−15.0 to −1.1) [February 2021]	−0.19 (−0.27 to −0.11)	−24.7 (−33.7 to 15.7)

^a^ Comprising stroke, myocardial infarction, congestive heart failure, transient ischemic attack, and angina episodes.

^b^ Presenting to the hospital.

^c^ Presenting to the emergency department, observation unit, or hospital.

Stroke hospitalizations initially decreased by 27.0% (95% CI, −39.5 to −14.5), remained lower than expected in February 2021 (−11.8%; 95% CI, −23.1 to −0.5), and were below expected levels in December 2021 (−27.3%; 95% CI, −42.4 to −12.2). Myocardial infarction hospitalizations initially decreased by 27.8% (95% CI, −35.8 to −19.8) but were not statistically different from expected by January 2021. Congestive heart failure episodes initially decreased by 26.1% (95% CI, −33.3 to −18.9), were below expected levels in March 2021 (−15.8%; 95% CI, −22.6 to −9.0), and were lower than expected by December 2021 (−22.1%; 95% CI, −31.5 to −12.7). Angina episodes followed similar sustained reduction trends, whereas TIA episodes were not statistically different from expected by August 2020.

## Discussion

The COVID-19 pandemic was not associated with longer-term increases in adverse CVEs among commercially insured New England residents presenting for care. Instead, we detected sustained reductions in TIA, CHF, and angina episodes. Factors explaining these trends could include lack of patient presentation during the 21-month follow-up, cardiovascular deaths outside the medical system, COVID-19–related deaths of people at risk for high-acuity CVEs, decrease in overdiagnosis due to lower emergency department and hospital volumes, heart failure management at home, and reductions in adverse events. Further studies are needed to identify and quantify such factors.

Study findings might not generalize to other US regions, people without commercial insurance, or populations with lower SES. For example, Massachusetts was unusual for experiencing no increase in cardiovascular deaths during the first 2 pandemic months.^6^ Additional research may examine regions with lower concentrations of clinicians, lower SES, and higher CVD burden. This study, combined with future studies, could help policymakers and insurers anticipate changes in major health outcomes during periods of limited health care access.

## Notes

Supplement 1.
eMethods
**eTable 1.** Population Characteristics in March 2017, 2020, and 2021 **eTable 2.** Population Characteristics in March 2017, 2020, and 2021 of Study Members from Connecticut, and Analogous Characteristics of the Overall Connecticut Population, Based on the 2014-2018 American Community Survey at the Census Tract Level **eTable 3.** Population Characteristics in March 2017, 2020, and 2021 of Study Members from Massachusetts, and Analogous Characteristics of the Overall Massachusetts Population, Based on the 2014-2018 American Community Survey at the Census Tract Level **eTable 4.** Population Characteristics in March 2017, 2020, and 2021 of Study Members from Maine, and Analogous Characteristics of the Overall Maine Population, Based on the 2014-2018 American Community Survey at the Census Tract Level **eTable 5.** Population Characteristics in March 2017, 2020, and 2021 of Study Members from New Hampshire, and Analogous Characteristics of the Overall New Hampshire Population, Based on the 2014-2018 American Community Survey at the Census Tract Level **eTable 6.** Billing Codes Used to Identify High-Acuity Cardiovascular Events
eReferences

Supplement 2.
Data Sharing Statement
