# Supplementary material for: Trends in High-Acuity Cardiovascular Events During the COVID-19 Pandemic
Source: JAMA Health Forum. 2024 Jan 5;5(1):e234572. doi: 10.1001/jamahealthforum.2023.4572 (PMC10770765; doi:10.1001/jamahealthforum.2023.4572)
Supplement: Supplement 1. — eMethods eTable 1. Population Characteristics in March 2017, 2020, and 2021 eTable 2. Population Characteristics in March 2017, 2020, and 2021 of Study Members from Connecticut, and Analogous Characteristics of the Overall Connecticut Population, Based on the 2014-2018 American Community Survey at the Census Tract Level eTable 3. Population Characteristics in March 2017, 2020, and 2021 of Study Members from Massachusetts, and Analogous Characteristics of the Overall Massachusetts Population, Based on the 2014-2018 American Community Survey at the Census Tract Level eTable 4. Population Characteristics in March 2017, 2020, and 2021 of Study Members from Maine, and Analogous Characteristics of the Overall Maine Population, Based on the 2014-2018 American Community Survey at the Census Tract Level eTable 5. Population Characteristics in March 2017, 2020, and 2021 of Study Members from New Hampshire, and Analogous Characteristics of the Overall New Hampshire Population, Based on the 2014-2018 American Community Survey at the Census Tract Level eTable 6. Billing Codes Used to Identify High-Acuity Cardiovascular Events eReferences [file jamahealthforum-e234572-s001.pdf]

Supplementary Online Content

Wharam JF, LeCates RF, Thomas A, et al. Trends in high-acuity cardiovascular events during the COVID-19 pandemic. *JAMA Health Forum.* Published online January 5, 2024. doi:10.1001/jamahealthforum.2023.4572

eMethods

eTable 1. Population Characteristics in March 2017, 2020, and 2021

eTable 2. Population Characteristics in March 2017, 2020, and 2021 of Study Members from Connecticut, and Analogous Characteristics of the Overall Connecticut Population, Based on the 2014-2018 American Community Survey at the Census Tract Level

eTable 3. Population Characteristics in March 2017, 2020, and 2021 of Study Members from Massachusetts, and Analogous Characteristics of the Overall Massachusetts Population, Based on the 2014-2018 American Community Survey at the Census Tract Level

eTable 4. Population Characteristics in March 2017, 2020, and 2021 of Study Members from Maine, and Analogous Characteristics of the Overall Maine Population, Based on the 2014-2018 American Community Survey at the Census Tract Level

eTable 5. Population Characteristics in March 2017, 2020, and 2021 of Study Members from New Hampshire, and Analogous Characteristics of the Overall New Hampshire Population, Based on the 2014-2018 American Community Survey at the Census Tract Level

eTable 6. Billing Codes Used to Identify High-Acuity Cardiovascular Events

eReferences

This supplementary material has been provided by the authors to give readers additional information about their work.

© 2024 Wharam JF et al. *JAMA Health Forum.*

eMethods

A. Creation of high-acuity cardiovascular event episodes

We created high-acuity cardiovascular event episodes by generating 10-day windows (beginning with the day of a high-acuity cardiovascular event visit to the outpatient or high-acuity setting) during which we captured visits that had the same cluster of relevant diagnoses (e.g., all stroke diagnoses; eTable 1). This approach prevents “double counting” of situations such as a 3-day hospitalization for myocardial infarction followed by a related emergency department visit 2 days later. We required that the diagnosis of interest be in the primary, secondary, or tertiary position. For myocardial infarction and stroke, we only included 10-day episodes if they included a hospitalization for the relevant diagnosis, a standard approach with high specificity.[1,2] For heart failure, angina, and transient ischemic attack, we included 10-day episodes if there was an emergency department, observation unit, or hospital diagnosis because these represent higher acuity presentations in contrast to outpatient-based care that might represent routine monitoring or follow-up of a preexisting condition or episode. Our measure of interest was the number of high-acuity episodes per patient per month presenting to the health system.

B. Age categories used to adjust monthly episode rates

We used the following age categories among patients aged 35 years and above: 35-39, 40-49, 50-54, 55-59 (referent), 60-64, 65-69, 70-74. 75-79, >=80.

C. Sample sizes and enrollment duration

Sample sizes ranged from 283,467 to 376,511 per month over the study period. Mean and median enrollment durations were 28.4 months and 24.0 months, respectively.

D. Statistical analysis

We hypothesized that over the follow up period, monthly points would initially be statistically below the trend predicted by on our 3-year baseline, and then be statistically above predicted in later months of the study period. Our data were organized at the person-month level, and subjects appeared only in months in which they were enrolled. Month rows in our analytic dataset were labelled 1 (March 2017, start of study/ baseline) to 58 (December 2021, end of study).

The cardiovascular measures of interest comprised the dependent variables in our statistical models, and the independent variables were age group, male/female, trend, level change, trend change, and change in trend change (i.e., trend change*trend change, a quadratic term). The trend term starts at 1 for the first month of the baseline and counts up to the last month (i.e., 58 in this case). The level change term is zero for all baseline months (i.e., months 1 to 36 in this case) and 1 for all follow up months (i.e., 38 to 58 in this case). The trend change term is zero for all baseline months then begins counting up from 1 (for the first follow up month) to the number of follow up months (i.e., 21 in this case). The quadratic term is the square of this trend change term.

We used related modeling approaches to (1) generate statistical estimates of absolute and relative differences versus expected at each month and (2) create unadjusted plots of predicted and fitted trends.

Statistical estimates of absolute and relative differences versus expected at each month

We first ran a linear segmented regression model with trend, level change, trend change, and trend change*trend change terms, adjusted for age category and sex, while accounting for person-level clustering. This was a least square regression without fixed effects and with standard errors robust to heteroskedasticity and within-cluster correlation. We specified the member ID as the clustering variable. This model produced an expected trend from April 2020 to December 2021 based on the March 2017 to February 2020 pre-COVID trend. It also fit a modeled trend to the follow-up points. Using this model and its parameter estimates, we applied nonlinear combinations of parameters to calculate each adjusted follow up point’s absolute and relative

© 2024 Wharam JF et al. *JAMA Health Forum.*

differences (and 95% confidence intervals) compared to the value predicted by the extension of the baseline linear trend.

Plot unadjusted predicted fitted trends

Because nonlinear combinations of parameters are unable to simultaneously generate adjusted parameter estimates and accurate fitted linear trends, we used a separate unadjusted model to create fitted and predicted trends for plotting and visualization. We used a linear segmented regression model with generalized estimating equations and we included trend, level change, trend change, and trend change*trend change terms, while accounting for person-level clustering. This model used a sandwich estimator for the standard errors.

© 2024 Wharam JF et al. *JAMA Health Forum.*

eTable 1. Population characteristics in March 2017, 2020, and 2021

	March 2017	March 2020	March 2021
Population size	361,341	293,709	295,082
Female	189,577	155,037	155,982
Age at index date, years			
35-39	46,899	37,530	37,956
40-49	103,898	76,540	75,450
50-54	62,737	46,163	45,916
55-59	63,768	51,080	50,221
60-64	57,097	48,614	49,683
65-69	17,763	19,447	20,411
70-74	5,818	7,971	8,634
75-79	1,936	3,485	3,774
80+	1,425	2,879	3,037
Census tract predominant race/ethnicity[1,2]			
Black	2,547	2,134	2,097
Hispanic	1,567	1,389	1,376
White	303,785	245,905	245,845
None	53,442	44,281	45,764
Living in lower-income census tract[1,3]	55,356	48,180	48,492
Living in lower-education census tract[1,4]	138,796	117,896	117,594
State of residence			
Connecticut	10,933	11,713	10,587
Massachusetts	251,705	189,855	190,395
Maine	34,976	40,787	42,185
New Hampshire	63,727	51,354	51,915

[1]Based on 2014-2018 American Community Survey at the census tract level. [2]Defined as >66% of members in the census tract identifying as the given race/ethnicity category. [3]Defined as >10% of households in the census tract living below the federal poverty level. [4]Defined as >35% of households in the neighborhood with high school or less education level. - [5]People with missing state of residence were included in analyses.

© 2024 Wharam JF et al. *JAMA Health Forum.*

**eTable 2.** Population characteristics in March 2017, 2020, and 2021 of study members from Connecticut, and analogous characteristics of the overall Connecticut population, based on the 2014-2018 American Community Survey at the census tract level				
	**Study Population (n)**			**State Overall Population (n)**[1]
	**March 2017**	**March 2020**	**March 2021**	**2014-2018**
Overall state population size	NA	NA	NA	3,581,504
Population size, ≥ age 35 years	10,933	11,713	10,587	2,038,860
Female	5,293	5,962	5,491	1079595
Age at index date, years				
35-39	1,361	1,472	1,441	210552
40-49	3,175	3,105	2,779	465659
50-54	2,073	2,154	1,845	272950
55-59	2,201	2,332	2,042	266170
60-64	1,639	2,078	1,930	235949
65-69	351	455	427	186091
70-74	105	78	84	141323
75-79	16	27	28	98877
80+	12	12	11	161289
Census tract predominant race/ethnicity[1,2,3]	4,292	4,840	4,247	
Black	62	63	67	32393
Hispanic	105	132	111	56205
White	8,527	9,062	8,206	2324139
None	2,239	2,456	2,203	1168767
Living in lower-income census tract[1,2,4]	1,729	1,926	1,672	861852
Living in lower-education census tract[1,2,5]	5,594	5,774	5,219	1807135

[1]Based on 2014-2018 American Community Survey at the census tract level. [2]Denominator for state-level characteristics is the overall state population rather than the age ≥35 years state population. [3]Defined as >66% of members in the census tract identifying as the given race/ethnicity category. [4]Defined as >10% of households in the census tract living below the federal poverty level. [5]Defined as >35% of households in the neighborhood with high school or less education level. [5]People with missing state of residence were included in analyses.

© 2024 Wharam JF et al. *JAMA Health Forum.*

eTable 3. Population characteristics in March 2017, 2020, and 2021 of study members from Massachusetts, and analogous characteristics of the overall Massachusetts population, based on the 2014-2018 American Community Survey at the census tract level

	**Study Population (n)**			**State Overall Population (n)[1]**
	**March 2017**	**March 2020**	**March 2021**	**2014-2018**
Overall state population size	NA	NA	NA	6,830,193
Population size, ≥ age 35 years	251,705	189,855	190,395	3,785,153
Female	133,003	101,051	101,340	2007095
Age at index date, years				
35-39	32,886	24,699	25,023	414820
40-49	72,979	50,314	49,592	880270
50-54	43,500	29,772	29,850	496392
55-59	43,204	32,410	32,107	481780
60-64	39,198	30,543	30,908	433667
65-69	13,306	12,728	13,191	349010
70-74	4,350	5,188	5,384	262745
75-79	1,334	2,282	2,370	177502
80+	948	1,919	1,970	288967
Census tract predominant race/ethnicity[1,2,3]	115,888	88,797	90,130	
Black	2,485	2,071	2,030	55120
Hispanic	1,462	1,257	1,265	148520
White	198,831	148,189	148,065	4869608
None	48,927	38,338	39,035	1756945
Living in lower-income census tract[1,2,4]	39,426	31,945	32,226	1796135
Living in lower-education census tract[1,2,5]	85,389	67,632	68,127	3118303

[1]Based on 2014-2018 American Community Survey at the census tract level. [2]Denominator for state-level characteristics is the overall state population rather than the age ≥35 years state population. [3]Defined as >66% of members in the census tract identifying as the given race/ethnicity category. [4]Defined as >10% of households in the census tract living below the federal poverty level. [5]Defined as >35% of households in the neighborhood with high school or less education level. [5]People with missing state of residence were included in analyses.

© 2024 Wharam JF et al. *JAMA Health Forum.*

eTable 4. Population characteristics in March 2017, 2020, and 2021 of study members from Maine, and analogous characteristics of the overall Maine population, based on the 2014-2018 American Community Survey at the census tract level

	Study Population (n)			State Overall Population (n)[1]
	**March 2017**	**March 2020**	**March 2021**	
Overall state population size	NA	NA	NA	1,332,813
Population size, ≥ age 35 years	34,976	40,787	42,185	812,501
Female	18,532	21,450	22,228	424787
Age at index date, years				
35-39	4,707	5,400	5,555	74321
40-49	10,020	10,840	11,041	167622
50-54	6,035	6,446	6,572	101508
55-59	6,540	7,708	7,751	108271
60-64	5,849	7,756	8,577	101153
65-69	1,302	1,796	1,814	89169
70-74	324	517	566	63259
75-79	113	187	171	43130
80+	86	137	138	63618
Census tract predominant race/ethnicity[1,2,3]	1,255	1,745	1,807	
Black	0	0	0	0
Hispanic	0	0	0	0
White	34,193	38,880	39,359	1321526
None	783	1,907	2,826	11287
Living in lower-income census tract[1,2,4]	7,551	8,744	8,983	375918
Living in lower-education census tract[1,2,5]	20,824	23,078	23,041	891252

[1]Based on 2014-2018 American Community Survey at the census tract level. [2]Denominator for state-level characteristics is the overall state population rather than the age ≥35 years state population. [3]Defined as >66% of members in the census tract identifying as the given race/ethnicity category. [4]Defined as >10% of households in the census tract living below the federal poverty level. [5]Defined as >35% of households in the neighborhood with high school or less education level. [5]People with missing state of residence were included in analyses.

© 2024 Wharam JF et al. *JAMA Health Forum.*

eTable 5. Population characteristics in March 2017, 2020, and 2021 of study members from New Hampshire, and analogous characteristics of the overall New Hampshire population, based on the 2014-2018 American Community Survey at the census tract level

	Study Population (n)			State Overall Population (n)[1]
	March 2017	March 2020	March 2021	
Overall state population size	NA	NA	NA	1,343,622
Population size, ≥ age 35 years	63,727	51,354	51,915	790,574
Female	32,749	26,574	26,923	408465
Age at index date, years				
35-39	7,945	5,959	5,937	76674
40-49	17,724	12,281	12,038	173040
50-54	11,129	7,791	7,649	106877
55-59	11,823	8,630	8,321	110487
60-64	10,411	8,237	8,268	95512
65-69	2,804	4,468	4,979	79605
70-74	1039	2188	2600	56110
75-79	473	989	1205	37173
80+	379	811	918	55096
Census tract predominant race/ethnicity[1,2,3]	6,015	5,018	5,148	
Black	0	0	0	0
Hispanic	0	0	0	0
White	62,234	49,774	50,215	1306244
None	1493	1,580	1,700	37378
Living in lower-income census tract[1,2,4]	6,650	5,565	5,611	192400
Living in lower-education census tract[1,2,5]	26,989	21,412	21,207	608561

[1]Based on 2014-2018 American Community Survey at the census tract level. [2]Denominator for state-level characteristics is the overall state population rather than the age ≥35 years state population. [3]Defined as >66% of members in the census tract identifying as the given race/ethnicity category. [4]Defined as >10% of households in the census tract living below the federal poverty level. [5]Defined as >35% of households in the neighborhood with high school or less education level. [5]People with missing state of residence were included in analyses.

© 2024 Wharam JF et al. *JAMA Health Forum.*

eTable 6. Billing codes used to identify high-acuity cardiovascular events

Cardiovascular event	International Classification of Diseases, 10th Edition, Codes
Stroke	G450, G451, G452, G46, G460, G461, G462, G463, G464, G465, G466, G467, G468, I60, I600, I6000, I6001, I6002, I601, I6010, I6011, I6012, I602, I6020, I6021, I6022, I603, I6030, I6031, I6032, I604, I605, I6050, I6051, I6052, I606, I607, I608, I609, I61, I610, I611, I612, I613, I614, I615, I616, I618, I619, I62, I629, I63, I630, I6300, I6301, I63011, I63012, I63013, I63019, I6302, I6303, I63031, I63032, I63033, I63039, I6309, I631, I6310, I6311, I63111, I63112, I63113, I63119, I6312, I6313, I63131, I63132, I63133, I63139, I6319, I632, I6320, I6321, I63211, I63212, I63213, I63219, I6322, I6323, I63231, I63232, I63233, I63239, I6329, I633, I6330, I6331, I63311, I63312, I63313, I63319, I6332, I63321, I63322, I63323, I63329, I6333, I63331, I63332, I63333, I63339, I6334, I63341, I63342, I63343, I63349<br>I6339, I634, I6340, I6341, I63411, I63412, I63413, I63419, I6342, I63421, I63422, I63423, I63429, I6343, I63431, I63432, I63433, I63439, I6344, I63441, I63442, I63443, I63449, I6349, I635, I6350, I6351, I63511, I63512, I63513, I63519, I6352, I63521, I63522, I63523, I63529, I6353, I63531, I63532, I63533, I63539, I6354, I63541, I63542, I63543, I63549, I6359, I636, I638, I639
Myocardial infarction	I21, I210, I2101, I2102, I2109, I211, I2111, I2119, I212, I2121, I2129, I213, I214, I219, I21A1, I21A9, I22, I220, I221, I222, I228, I229
Congestive heart failure	E877, E8770, E8779, I0981, I110, I130, I132, I50, I501, I502, I5020, I5021, I5022, I5023, I503, I5030, I5031, I5032, I5033, I504, I5040, I5041, I5042, I5043, I50810, I50811, I50812, I50813, I50814, I5082, I5084, I5089, I509
Angina	I20, I200, I201, I208, I209, I24, I240, I248, I249, I2511, I25110, I25111, I25118, I25119, I257, I2570, I25700, I25701, I25708, I25709, I2571, I25710, I25711, I25718, I25719, I2572, I25720, I25721, I25728, I25729, I2573, I25730, I25731, I25738, I25739, I2575, I25750, I25751, I25758, I25759, I2576, I25760, I25761, I25768, I25769, I2579, I25790, I25791, I25798, I25799
Transient ischemic attack	G45, G453, G458, G459, I6781, I6782, I67848

© 2024 Wharam JF et al. *JAMA Health Forum.*

eReferences

1. Validity of Claims-Based Stroke Algorithms in Contemporary Medicare Data | Circulation: Cardiovascular Quality and Outcomes. Accessed June 30, 2023. https://www.ahajournals.org/doi/full/10.1161/CIRCOUTCOMES.113.000743

2. Kiyota Y, Schneeweiss S, Glynn RJ, Cannuscio CC, Avorn J, Solomon DH. Accuracy of medicare claims-based diagnosis of acute myocardial infarction: estimating positive predictive value on the basis of review of hospital records. *American Heart Journal*. 2004;148(1):99-104. doi:10.1016/j.ahj.2004.02.013

© 2024 Wharam JF et al. *JAMA Health Forum.*
