# Supplementary material for: Trends in High-Acuity Cardiovascular Events During the COVID-19 Pandemic
Source: JAMA Health Forum. 2024 Jan 5;5(1):e234572. doi: 10.1001/jamahealthforum.2023.4572 (PMC10770765; doi:10.1001/jamahealthforum.2023.4572)
Supplement: Supplement 2. — Data Sharing Statement [file jamahealthforum-e234572-s002.pdf]

Data Sharing Statement

Wharam. Trends in High-Acuity Cardiovascular Events During the COVID-19 Pandemic. *JAMA Health Forum*. Published January 05, 2024. doi:10.1001/jamahealthforum.2023.4572

Data
Data available: No

Additional Information
Explanation for why data not available: Not allowed by data partner
